# Fucoidan-Based Nanoparticles with Inherently Therapeutic Efficacy for Cancer Treatment

**DOI:** 10.3390/pharmaceutics13121986

**Published:** 2021-11-23

**Authors:** Chih-Sheng Chiang, Bo-Jie Huang, Jui-Yu Chen, Wee Wei Chieng, Seh Hong Lim, Wei Lee, Weoi-Cherng Shyu, Long-Bin Jeng

**Affiliations:** 1 Graduate Institute of Biomedical Sciences, China Medical University, Taichung 404, Taiwan; 2 Cell Therapy Center, China Medical University, Taichung 404, Taiwan; 3 Neuroscience and Brain Disease Center, China Medical University, Taichung 404, Taiwan; 4 Translational Medicine Research Center, China Medical University, Taichung 404, Taiwan; bojie@nanoriginal.onmicrosoft.com (B.-J.H.); juiyu0314@nanoriginal.onmicrosoft.com (J.-Y.C.); mcsherry@nanoriginal.onmicrosoft.com (W.W.C.); T24266@mail.cmuh.org.tw (W.L.); 5 Department of Physician Assistant Studies, MGH Institute of Health Professions, Boston, MA 02129-4557, USA; chrislim@mghihp.edu; 6 Department of Neurology, China Medical University, Taichung 404, Taiwan; 7 Department of Occupational Therapy, Asia University, Taichung 404, Taiwan; 8 Organ Transplantation Center, China Medical University, Taichung 404, Taiwan

**Keywords:** fucoidan nanoparticle, marine polysaccharide, safety, antitumor effect, anti-metastasis, parenteral administration

## Abstract

The anticancer properties of fucoidan have been widely studied in cancer research. However, the lack of safety information on the parenteral administration of fucoidan and its rapid clearance from the system have limited its application. Herein, we assessed the therapeutic efficacy and safety of fucoidan and developed fucoidan nanoparticles (FuNPs) to enhance their therapeutic effect in the mouse model of breast cancer. FuNPs were synthesized through the emulsion method, and the stable colloid has an average size of 216.3 nm. FuNPs were efficiently internalized into breast cancer cells in vitro, demonstrating an enhanced antitumor activity in comparison with free form fucoidan. After the treatment of FuNPs, the tumor progression was significantly inhibited in viv. The tumor volume was reduced by 2.49-fold compared with the control group. Moreover, the inhibition of the invasion of tumor cells into the lungs revealed the antimetastatic properties of the FuNPs. FuNPs, as naturally marine polysaccharide-based nanoparticles, have shown their broader therapeutic window and enhanced antimetastatic ability, opening an avenue to the development of the inherently therapeutic nanomedicines.

## 1. Introduction

Fucoidan is a fucose-containing sulphated polysaccharide extracted from brown seaweeds. The biological functions of this marine polysaccharide include anticancer, antivirus, anti-coagulant, and modulation on diabetic and metabolic syndrome, have been extensively studied [1]. With advantages such as being high solubility in aqueous solution and a favorable safety profile, fucoidan has been applied to cancer treatments in preclinical and clinical studies [2,3,4].

The therapeutic effects of fucoidan have been demonstrated in abundant cancer types including acute leukemia [5], lymphoma [6], head and neck [7], lung [8], breast [9], hepatoblastoma [10], prostate [11], and ovarian cancer [10] in animal studies. The mechanisms or reactions discovered in these cancers were mostly associated with the induction of apoptosis and the inhibition of cell cycle. Moreover, fucoidan possesses antimetastatic ability [12], inhibiting the formation of tumor nodules in the lungs of metastatic 4T1 tumor-bearing animal model [9]. Fucoidan has also been used as a complementary therapy for patients in clinical studies, and the results demonstrated that fucoidan may augment the therapeutic index [2].

In current applications, fucoidan is mostly administered via oral delivery. However, obstacles such as the relatively low bioavailability [13] and the fast clearance rate [14] have restricted the effective accumulation of fucoidan within the tumor, and thus limited its therapeutic efficacy. Engineered nanoparticles with the tailorable properties such as size, zeta potential, surface coating, and shape can optimize the pharmacokinetic behavior and the ability of one material to accumulate in a tumor [15,16]. As reported by Abdollah et al., the coating of fucoidan on the surface of nanoparticles can significantly extend its circulation time in the bloodstream [17], thus offering more opportunities for the nanomedicines to accumulate within the tumor.

In this study, we developed a fucoidan-based nanoparticle (FuNP), explored its safety profile and its potential to inhibit tumor progression via parenteral administration route. Herein, we synthesized the colloidally stable FuNPs using the emulsion method. The compositions we used to stabilize the structure are listed as approved excipients by USFDA. The safety profiles of fucoidan and FuNPs injected via parenteral route were evaluated in mice, and the therapeutic efficacy of FuNPs was assessed in MDA-MB-231-tumor bearing mice. We demonstrated that FuNPs possessed a favorable safety profile and the potency to inhibit tumor progression as well as metastasis. This evidence paves the way for further translational applications of FuNPs.

## 2. Materials and Methods

### 2.1. Materials and Reagents

Poly(d,l-lactide-co-glycolide) (PLGA), soybean oil, and Poly(lactide-co-glycolide)-Rhodamine B were purchased from Merck (Germany). Anti-F-actin antibody and 2-(4-Amidinophenyl)-6-indolecarbamidine dihydrochloride, 4′,6-Diamidino-2-phenylindole dihydrochloride (DAPI) were purchased from BD Biosciences (USA). Fucoidan from fucus vesiculosus was purchased from Marinova (Tasmania, Australia).

### 2.2. Preparation of Fucoidan Nanoparticles (FuNPs)

FuNPs were synthesized by emulsification process. The aqueous phase with fucoidan and organic phase (i.e., dichloromethane, DCM) with PLGA plus soybean oil were mixed and sonicated using the ultrasonic homogenizer (UP200S with S2 probe, Hielscher, Germany) under ice bath. After forming a one-phase emulsion, the sample was subjected to the rotary evaporator to remove the DCM. The aqueous solution after evaporation was purified using a centrifuge.

### 2.3. Size Selection of FuNPs Using Centrifuge

The as-synthesized FuNPs were subjected to size selection to separate the large and small FuNPs. The group without the exclusion of large FuNPs was centrifuged at 14,000× *g* for 15 min. We then removed the suspension and redispersed them in water. To collect the large FuNPs, the as-synthesized FuNPs were centrifuged at 800× *g* for 5 min. The pellets represented the larger FuNPs were collected and redispersed in water, while the suspension was further centrifuged at 14,000× *g* for 15 min. The pellet as the smaller FuNPs was redispersed in water and stored in 4 °C for further use.

### 2.4. Characterization of FuNPs

The morphology and structure of FuNPs were characterized using a scanning electron microscopy (SEM, JSM-6700F, JOEL, Akishima, Japan) and a transmission electron microscopy (TEM, JEOL JEM-2100F, JEOL, Akishima, Japan). Dynamic light scattering was used (DLS, Litesizer 500, Anton Paar, Graz, Austria) to measure the size and zeta potential of FuNPs.

### 2.5. Stability Test

FuNPs were transferred from water to PBS for the stability test. In brief, the FuNPs were stored in 4 °C and 37 °C, respectively, and the size and zeta potential of the FuNPs were analyzed at least 3 times for 7 days post PBS incubation.

### 2.6. Cell Culture

MDA-MB-231 breast cancer cell line was purchased from Bioresource Collection and Research Center, Food Industry Research and Development Institute, Taiwan. The cells were cultured using Dulbecco’s Modified Eagle Medium (DMEM, Thermo Fisher Scientific, MA, USA) containing 10% fetal bovine serum (FBS, from Thermo Fisher Scientific, MA, USA) and 1% penicillin/streptomycin (Thermo Fisher Scientific, MA, USA). The 4T1 was purchased from ATCC (Manassas, Virginia, USA), and the culture method was identical to MDA-MB-231 cells.

### 2.7. In Vitro Antitumor Effects

MDA-MB-231 cells were treated with fucoidan or FuNPs at different concentrations and the cell viabilities at 24 h and 48 h were measured using CCK-8 assay. In brief, 5 × 10^3^ cells were seeded in 96 well plates and cultured with culture medium (100 μL) at 37 °C overnight. Fucoidan and FuNPs with different concentrations were then added to the cells. After incubation for 24 h or 48 h, cells were washed with PBS, and CCK-8 solution was added to the medium and allowed reaction for 30 min. The plate was then subjected to Elisa reader (SpectraMax iD3, Molecular Devices, San Jose, CA, USA) and the absorbance was measured at 450 nm.

### 2.8. Monitoring Cell Internalization

We analyzed the cell internalization using flow cytometry (Attune Nxt flow cytometry, ThermoFisher, USA) and fluorescence microscopy (AxioImager. A1, Zeiss, Germany). To facilitate the observation, PLGA-Rhodamine was mixed with PLGA at 1:20 *w*/*w* ratio during the synthesis process to obtain the fluorescence-labeled FuNPs. After the incubation of FuNPs with 4T1 and MDA-MB-231 cells for 24 h and 48 h in a 6-well plate, the cells (1 × 10^5^) were collected and measured using flow cytometry.

For fluorescence microscopy, 4T1 cells were seeded and allowed to grow on Millicell EZ SLID (Merck, Germany) overnight. After that, we incubated the cells with FuNPs for 24 h and washed the cells twice with PBS. To generate cellular permeability, triton X-100 (0.3% in PBS buffer) was added for 30 min. The cells were then blocked using FBS buffer (30 min), immobilized using anti-F-actin at 4 °C overnight, and further stained using a secondary antibody (1:250) for 1 h. After an 1 h incubation, the cells were washed twice with PBS and stained with DAPI (1 µg mL^−1^) for 5 min. The cells were then observed using a fluorescent microscope.

### 2.9. Animals

ICR mice were used in the safety study, in which the animal protocol has been reviewed and approved by the Institute of Animal Care and Use Committee (IACUC) of Agriculture Technology Research Institute, Taiwan, approval number of 109113. For therapeutic and safety studies using balc/c nude mice, the animal experiments were performed in compliance with the guidelines of the Animal Care and Use Committee (IACUC) of the China Medical University under the approval number of CMUIACUC-2021-299.

### 2.10. Safety of Fucoidan via IP Injection

A 14-day acute toxicity study of fucoidan via intraperitoneal (IP) injection was carried out to determine the safety and the adverse effect of fucoidan on 12 male and 12 female adult ICR mice (7–9 weeks old). Mice were randomly assigned to either Control, Low-dose, Mid-dose or High-dose groups in which they intraperitoneally (IP) received a 0, 50, 275 or 500 mg kg^−1^ of single dose fucoidan, respectively. During the 14-day observation period, clinical observation including body weight, mortality, clinical symptoms including exterior, behavior, breath, mouth and nose, eyes, skin, digestion, and metabolism were monitored and recorded. Following the monitoring for 14 days, the mice were sacrificed and were subjected to assessments including hematology, serum chemistry and histopathology. For hematology, the whole blood was collected from abdominal aorta through syringe aspiration and preserved in clean K^2^-EDTA centrifuge tubes at room temperature before the analysis of red blood cells (RBC), hemoglobin (HGB), white blood cells (WBC), hematocrit (HCT), blood platelet (PLT), lymphocyte composition, eosinophil composition, neutrophil composition, monocyte composition, and basophil composition.

For serum chemistry, the whole blood collected from the heart through syringe aspiration was preserved in clean centrifuge tubes at room temperature for 30 min and centrifuged to harvest the serum. Aspartate aminotransferase (AST), alanine aminotransferase (ALT), creatinine, blood urea nitrogen (BUN), albumin, and glucose of the mice were measured using clinical chemistry analyzer.

For histopathology, the harvested organs include adrenal glands, heart, lungs with trachea, kidneys, spleen, liver with gallbladder, salivary glands, and mandibular lymph were preserved in 10% neutral buffered formalin (NBF) at room temperature for 72~96 h. After fixation, the tissues were trimmed, dehydrated with ethanol and infiltrated by paraffin. Thin sections (4–6 μm in thickness) were cut using a microtome and were subjected to hematoxylin and eosin (H&E) stain. Histopathological alterations were documented by a pathologist.

### 2.11. Therapeutic Effect of FuNPs

To evaluate the therapeutic effect, the MDA-MB-231 tumor-bearing mice were treated with saline (control) or FuNPs via intravenous (i.v, 100 mg kg^−1^) injection (q3dx6) at 19 days after tumor inoculation (*n* = 4). The tumor volumes were monitored using a digital caliper (Mitutoyo) three times a week using the following Equation (1):

(1) $$\mathrm{Tumor}\;\mathrm{volume}\;(V)=\frac{W\times L^{2}}{2}$$

in which W is the width of the tumor, and the L is the length of the tumor (W < L).

### 2.12. Safety of FuNPs

Following the monitoring of the tumor progression, the mice were later sacrificed on day 49 and were subjected to histopathological assessments. In brief, the harvested organs include adrenal glands, heart, lungs with trachea, kidneys, spleen, liver with gallbladder, salivary glands, mandibular lymph node, and inoculated neoplasm (human breast cancer model, MDA-MB-231 cell line) were preserved in 10% neutral buffered formalin (NBF) at room temperature for 72~96 h. After fixation, the tissues were trimmed, dehydrated with ethanol, and infiltrated by paraffin. The microtome was used to cut the sample with multiple sections with 4–6 μm in thickness These sections were subjected to hematoxylin and eosin (H&E) stains to observe histological alteration by the study pathologist.

### 2.13. Statistical Analysis

Results are expressed as mean ± s.d. unless otherwise noted. Non-parametric Kruskal–Wallis test was used to evaluate the significance of mean differences between the control and the fucoidan-treated group in body weights and clinical chemistry values. One-way ANOVA was used to evaluate the statistical differenceusing GraphPad Prism version 9.2.0, USA. *p* value < 0.05 was considered statistically significant. If a significant result was determined, post hoc tests were used to seek the differences with the control group.

## 3. Results

### 3.1. Safety of Fucoidan

The high biocompatibility of fucoidan has been recognized by the Generally Recognized as Safe (GRAS) of the Food and Drug Administration (FDA). While most studies focus on the safety of fucoidan using the oral administration route, scarce information on the dose-related toxicity via parenteral administration has been revealed.

**Figure 1:**
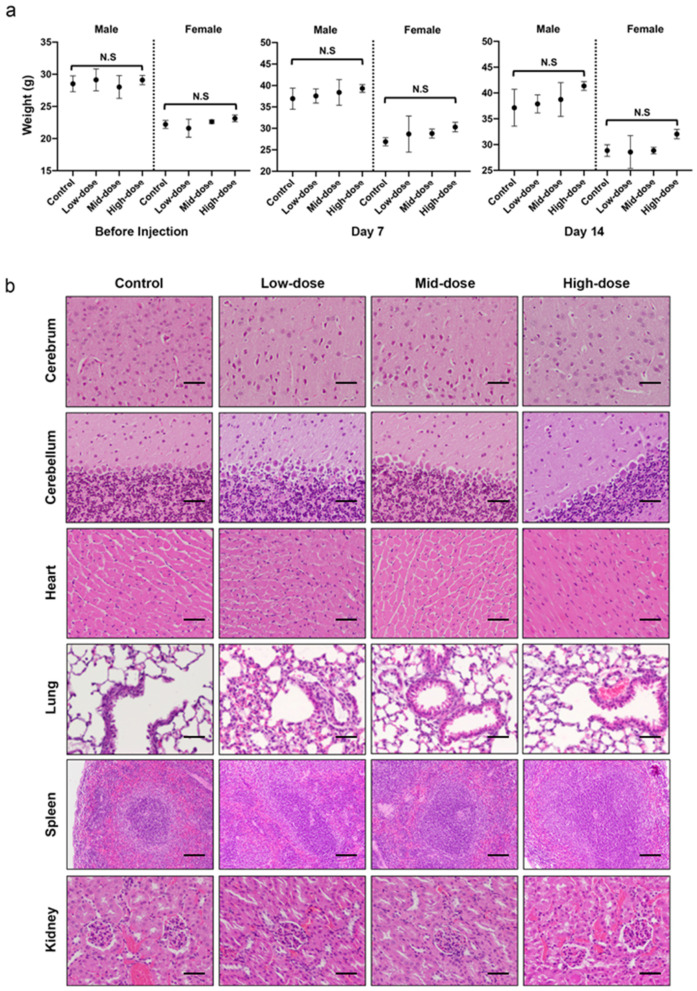
The use of fucoidan at the single dose of 50–275 mg kg^−1^ via intraperitoneal injection is determined safe in mice. (**a**) Left panel: there was no significant difference in body weight of the males and females when they were randomly assigned to each group before the injection. Middle panel: The body weight of the mice was not affected 14 days after the exposure to a single dose of Fucoidan. Right panel: Low-, mid- and high-dose Fucoidan did not affect the body weight of the mice at the end point of the toxicity test. (**b**) Representative microscopic images depict no observable impact of fucoidan to the brain, heart, lung, spleen, and kidney. All data are shown in means ± s.d. Exact *p*-values are reported in text. Scale bar = 50 μm for cerebrum, cerebellum, heart, lung, and kidney = 100 μm for spleen.

Herein, a 14-day acute toxicity study of fucoidan via intraperitoneal (IP) injection was carried out to determine the safety and the adverse effect of fucoidan on 12 male and 12 female adult ICR mice (7–9 weeks old). Mice were randomly assigned to either Control, Low-dose, Mid-dose or High-dose groups in which their IP received a 0, 50, 275 or 500 mg kg^−1^ of single dose fucoidan, respectively. All mice survived to the end of the study without any abnormal clinical manifestation on their skin, eyes, nose, and mouth. They exhibited normal breathing, behavioral, digestion and metabolism patterns. There was no statistical difference in body weight across all groups before the injection, 7 days (Kruskal–Wallis; *p* = 0.514 for male, *p* = 0.182 for female) and 14 days (Kruskal–Wallis; *p* = 0.304 for male, *p* = 0.227 for female) after the IP injection of fucoidan (Figure 1a). Both the clinical chemistry (Supplementary Table S1a) and hematology values (Supplementary Table S1b) of all groups were within normal reference range [18]. Histopathological examination revealed that cerebrum, cerebellum, heart, lung, spleen, and kidney remained healthy and normal across groups (Figure 1b) except for liver and lymphatic organs.

Their abnormalities were only to be observed in the High-dose group. The abnormal histopathological findings were observed in multiple lymphatic organs. Interestingly, when comparing with the Control, Low-dose and Mid-dose groups, the thymus of the High-dose group was significantly heavier (Supplementary Figure S1) and demonstrated a mild increase of corticomedullary ratio (Supplementary Figure S2a). Mild to moderate hyperplasia were found in gut-associated lymphoid tissue (GALT, Supplementary Figure S2b) and mesenteric lymph nodes, mostly in their follicular (Supplementary Figure S2c), indicating the immune enhancement effect at this area was dominated by T cells. Besides the lymphatic organs, the males’ livers were weighted heavier in the High-dose group than those in the Control, Low-dose and Mid-dose groups (Supplementary Figure S1). In addition, mild to moderate hepatic lesions along with microvascular foamy microphages aggregation were found only in some of the female mice in the High-dose group (Supplementary Figure S2d). Based on the result of this acute toxicity test, the decision was made to the dose below Mid-dose (i.e., 275 mg/kg) in the subsequent studies to minimize the interference of adverse effects.

### 3.2. Characterization of Fucoidan Nanoparticles (FuNPs)

**Figure 2:**
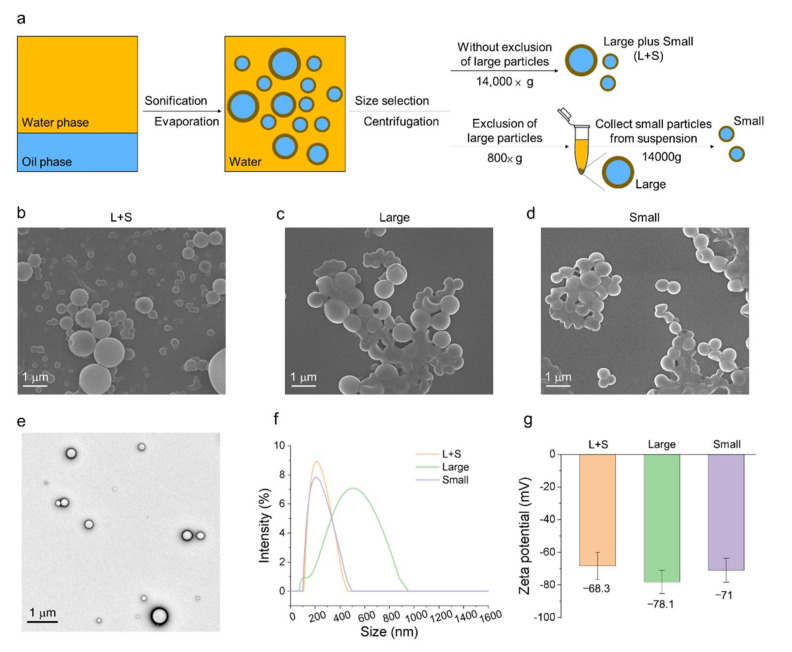
Illustration of the size selection strategy and the characterization of FuNPs. (**a**) Schematic illustration of size selection for FuNP using centrifugation. SEM images of L+S (**b**), Large (**c**), and Small (**d**) FuNPs groups; (**e**) TEM image of the Small FuNPs group; The size (**f**) and zeta potential (**g**) of the different groups were measured using DLS.

We synthesized FuNPs using the emulsion method. The size and the structural stability of FuNPs can be controlled by adjusting the composition, solvent, and sonication parameters including the intensity and the process time. Although emulsion is a well-established method to synthesize nanoparticles, the size distribution of the produced nanoparticles is broad. The size of a nanoparticle affects its interaction, internalization, and degradation with a cell, which would consequently impact its heterogenous performance on efficacy and safety [15]. Therefore, after emulsification, we performed size selection using the centrifugation and the selection strategy is illustrated in Figure 2a. The FuNPs without exclusion of large particles were abbreviated as L+S, while the FuNPs obtained via size selection process were categorized into either “Large” or “Small” groups.

The sizes of the FuNPs were then verified using scanning electron microscopy (SEM), transmission electron microscopy (TEM), and dynamic light scattering (DLS). Heterogeneous size distribution of the L+S group was observed (Figure 2b). After size selection, the homogeneous FuNPs of Large (Figure 2c) and Small groups (Figure 2d) were observed using SEM. They were further analyzed and verified using DLS (Figure 2f). The size of the FuNP in the L+S, Large, and Small groups were 223.6, 410.1, and 216.3 nm, respectively, indicating the vast majority of the produced FuNPs were in the smaller size. The homogenous FuNPs in the Small group were then observed using TEM (Figure 2e). These FuNPs were strongly negatively charged due to the sulphate groups present in the fucoidan structure (Figure 2g). FuNPs with larger size demonstrated a relatively stronger charge, possibly due to the fact that more fucoidan molecules were coated on the surface.

### 3.3. The Effect of the Size of Fucoidan Nanoparticles on Cell Uptake

The size of a nanoparticle can affect the efficiency of cellular interaction and internalization. After breast cancer cell line 4T1 and MDA-MB-231 were incubated with the FuNPs from L+S, Large, and Small groups for 24 h or 48 h, the cell internalization efficiency was assessed using flow cytometry. Rhodamine-modified PLGA were introduced in the emulsion process to form the dye-labeled FuNPs to facilitate the observation.

**Figure 3:**
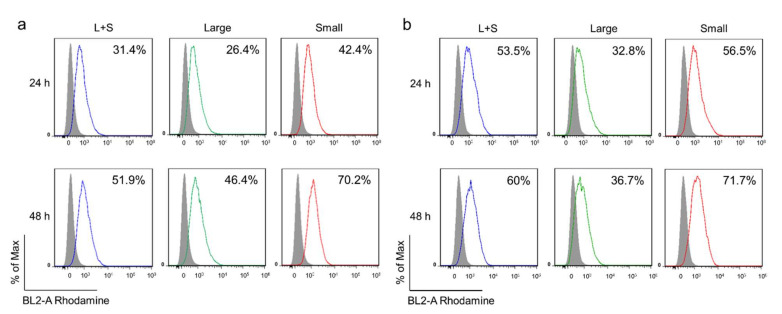
Internalization of FuNPs with different sizes into cancer cell lines. The percentage of 4T1 (**a**) and MDA-MB-231 cells (**b**) internalizing FuNPs from L+S, Large, and Small groups at 24 h and 48 h incubation was evaluated using flow cytometry.

The percentage of the cells that internalized FuNPs increased following the time-dependent manner in both cells. At 24 h incubations, 31.4%, 26.4%, and 42.4% of the 4T1 cells, and 53.5%, 32.8%, and 56.5% of the MDA-MB-231 cells treated with L+S, Large, and Small FuNPs, respectively, were found to associate with the FuNPs (Figure 3). FuNPs from L+S and Small groups demonstrated a higher fluorescent intensity compared with the Large group, indicating that the smaller FuNPs possessed a higher cell internalization efficiency compared with the larger ones. FuNPs from the Small groups also exhibited a higher cell internalization efficiency compared with those of L+S and Large groups at 48 h post incubation in both cells (Figure 3). These results were confirmed using a fluorescent microscope. The higher red fluorescence intensity (rhodamine-labeled FuNPs) was found in the cancer cells for the Small group (Supplementary Figure S3). We also found that FuNPs from the Large group were less stable and tended to precipitate in PBS and culture media after 24 h of incubation. In contrast, the Small group was colloidally stable during the assessments, which might provide a higher opportunity to interact with the cells to facilitate cellular internalization. Thus, FuNPs from the Small group were chosen to perform subsequent in vitro and in vivo studies.

### 3.4. Stability of the FuNPs

**Figure 4:**
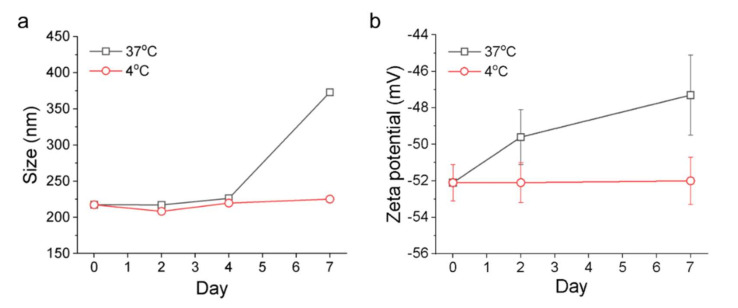
Monitoring the stability of FuNPs in PBS by size (**a**) and zeta potential (**b**) using DLS from day 0 (as synthesis) to day 7.

The stability of FuNPs were assessed by incubating them in PBS at 4 °C and 37 °C, respectively. FuNPs at 4 °C were found to be stable in PBS, demonstrating identical size (Figure 4a) and zeta potential (Figure 4b) from day 0 to day 7. The polydispersity index (PDI) at 0.05, 0.02, and 0.012 for 2-, 4-, and 7-day incubation demonstrated that FuNPs sustained homogenous size distribution. Of note, since the ion environment in PBS is more complex, the zeta potential of FuNPs measured in this experiment was higher than the value in water (Figure 2g). On the other hand, at 37 °C, the size of FuNPs increased significantly on day 7 and the zeta potential of FuNPs increased significantly at day 2 and day 7. These changes happened probably due to the degradation of PLGA.

### 3.5. Cytotoxicity of Fucoidan and FuNPs on Cancer Cells In Vitro

**Figure 5:**
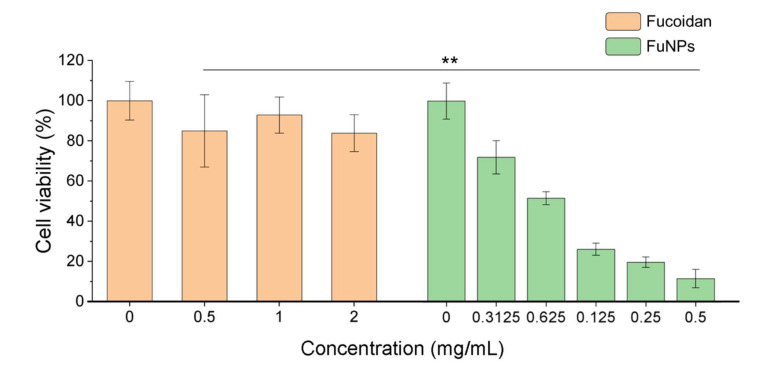
The cell viability of MDA-MB-231 after treating fucoidan or FuNPs with various concentrations. The experiments were performed with at least 6 biological independent samples. One-way ANOVA was performed to evaluate the statistical difference of cell viability between the cells treated with fucoidan and FuNPs at the concentration of 0.5 mg mL^−1^; ** *p* < 0.01.

To evaluate the cytotoxicity of fucoidan and FuNPs, different concentrations were used to treat MDA-MB-231 cells. Interestingly, we found that FuNPs showed stronger antitumor effect while compared with the fucoidan (Figure 5). MDA-MB-231 cells sustained a viability of more than 80% in the exposure to fucoidan (0.5 to 2 mg mL^−1^). In contrast, the FuNPs significantly decreased the viability of MDA-MB-231 cells at a concentration as low as 0.3125 mg mL^−1^. At 0.5 mg mL^−1^, cell viability was approximately 7 times lower when treated with FuNPs than with fucoidan, suggesting the anticancer potency of FuNPs was significantly stronger than free form fucoidan. Based on its superior efficacy in cytotoxicity studies, FuNPs were assessed in the MDA-MB-231 animal model to further evaluate its anticancer effects.

### 3.6. Tumor Inhibition Effect

**Figure 6:**
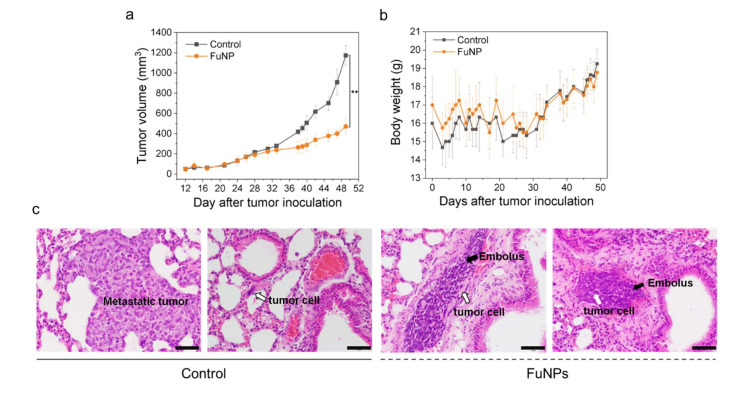
Therapeutic efficacy of FuNPs in MDA-MB-231-tumor bearing mice. Tumor volume (**a**) and body weight change (**b**) of the MDA-MB-231-tumor-bearing mice treated with saline (control) and FuNPs following the treatment course of q3dx6; the section of lung in control and FuNPs-treated groups. (**c**) H&E stains of the mice’s lungs showed metastatic tumors in the control group, while the invasion was inhibited in the FuNPs-treated group. Scale bar = 50 μm. The experiments were performed with 4 biological independent animals. One-way ANOVA was performed in Figure 6a to evaluate the statistical difference between the tumor volume at day 49.

FuNPs demonstrated therapeutic effect in MDA-MB-231 tumor-bearing mice in a q3dx6 treatment regimen, showing a superior tumor inhibition effect compared with the control group (*p* = 0.003, day 49, Figure 6a). The body weight of the mice treated with FuNPs slowly increased over time, showing no significant difference in comparison with the control group (Figure 6b). The results indicated that the administration of FuNPs inhibited the growth of the MDA-MB-231 tumor, while no abnormal clinical manifestation was found throughout the treatment.

On day 49 post tumor inoculation, the mice were sacrificed, and the major organs were subjected to pathological analysis. The lungs of the mice treated with saline (control groups) were found to be invaded by the metastatic tumor cells (Figure 6c). Importantly, the sections of the arterial lumen of the FuNPs-treated groups were dominated with embolus, which comprised spindle cells, fibrin, immune cells, and tumor cells. This evidence was a heritage that the tumor cells failed to extravasate into lung parenchyma, thus preventing the tumor metastasis (Figure 6c).

### 3.7. Safety of Fucoidan Nanoparticles

**Figure 7:**
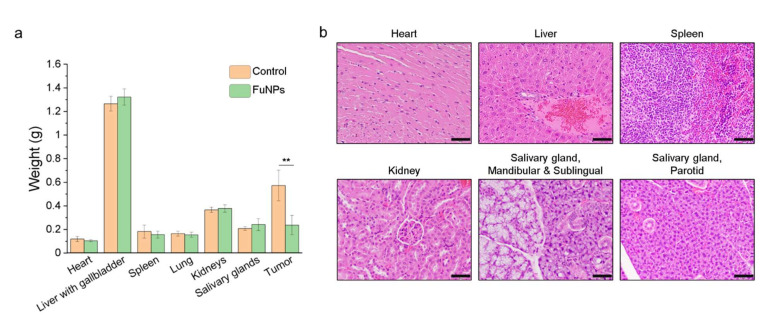
Safety assessments of FuNPs. (**a**) The organs and tumors weight of the mice treated with saline (control) and FuNPs; (**b**) H&E stain for the pathological analysis of the mice treated with FuNPs. Scale bar = 50 μm. The experiments were performed with 4 biological independent animals. One-way ANOVA was performed in Figure 7a to evaluate the statistical difference between the tumor weight between control and FuNP-treated mice; ** *p* < 0.01.

After sacrificing the mice, the organs including hearts, livers with gallbladders, spleens, lungs, kidneys, and salivary glands as well as tumors were collected, weighted, sectioned, stained, and subjected to pathological assessments. There were no significant differences in the organs from the mice treated with saline and FuNPs (Figure 7a). As the tumor growth was effectively inhibited as shown in Figure 6a, the average weight of the tumor from the mice treated with FuNPs was significantly lighter compared with the control group (Figure 7a). We did not observe any abnormality from the H&E stains of heart, liver, spleen, kidney, and salivary gland of the mice treated with FuNPs (Figure 7b). With all the evidence, we concluded that FuNPs are safe under current treatment regimen and dose.

## 4. Discussion

Fucoidan from fucus vesiculosus was demonstrated to be safe at the doses ranging from 50 to 500 mg kg^−1^ and 50 to 275 mg kg^−1^ via the parenteral IP administration route in male and female mice, respectively (Figure 1 and Supplementary Figure S1). While not every female mouse that received 500 mg kg^−1^ fucoidan showed liver lesion, further studies involving more female mice are required to draw conclusions. Generally, our study provides solid evidence that fucoidan is a substance with a favorable safety profile for the applications associated with parenteral administration.

With the simple emulsion process, we synthesized the FuNPs with an inherently therapeutic effect on tumor inhibition. In Figure 2a–d, we showed that larger FuNPs could be collected via centrifugation at a lower speed (i.e., 800× *g*). By excluding the FuNPs with the particle size larger than 400 nm, the smaller ones presented a more efficient cell internalization efficacy in MDA-MB-231 and 4T1 breast cancer cell lines (Figure 3). The storage of FuNPs in PBS at 4 °C was demonstrated to be stable for at least one week. In contrast, zeta potential and size of FuNPs significantly increased at 2- and 7-day post incubation in PBS at 37 °C (Figure 4), indicating the structural instability took place within 48 h when subjecting the environment. The instability might be attributed to the hydrolytic degradation of PLGA in aqueous environment [19], and lead to the detachment of fucoidan from the particle surface. The degradation of FuNPs at 37 °C (i.e., the biological temperature) addresses the potential of FuNP being a vesicle to encapsulate, deliver, and release active pharmaceutical ingredients to a tumor tissue, and such controlled drug release application is worth exploring in the future studies.

The anti-cancer activity and biological effects of fucoidan have been studied extensively. The regulation of cell cycle, inhibition for the receptors such as epidermal growth factor receptor (EGFR), transforming growth factor-beta receptors (TGFF), and Toll-like receptor 4 (TLR4) for triggering downstream apoptotic pathways [2,20] are among the common mechanisms that fucoidan activates to suppress the proliferation of cancer cells. Noteworthily, FuNPs we synthesized had a significantly stronger anti-cancer activity compared with the free form fucoidan (Figure 5). The stronger anti-cancer activity could be attributed to the nanoparticle-related factors such as the enhanced cell uptake efficiency and the induction of higher-level reactive oxygen species (ROS) in cancer cells [21].

FuNPs significantly inhibited the tumor progress in MDA-MB-231-bearing mice, indicating the nanoscale formulation can successfully accumulate in the tumors after being IV injected into the bloodstream (Figure 6a). Importantly, fucoidan has been demonstrated to inhibit metastasis in multiple cancer types including breast, lung, and liver cancer [12,20]. Encouragingly, FuNPs also demonstrated antimetastatic activities, where the tumor cells were found to lose the ability to invade into the lung after receiving the FuNPs treatments (Figure 6c). In our study, multiple doses were administered in a 3-day treatment plan, and no adverse effect was observed in the histological assessments (Figure 7). This indicates that FuNPs are a safe formulation with the dual effects of inhibiting tumor growth and metastasis under the current treatment regimen and dose. A further exploration of FuNPs dose escalation study is valuable since FuNPs have such a favorable therapeutic index and broad safety margin.

The aim of this study is to explore the possibility of forming fucoidan-based nanoparticles and evaluate their anti-cancer activity. Our results demonstrated that the stable FuNPs with appropriate size have a far superior tumor progress inhibition ability. The anti-metastasis function of FuNPs was also observed in the mouse cancer model. Noteworthily, under the intensive treatment regimen, FuNPs did not induce adverse effects as confirmed by histological analysis. However, future studies elucidating the mechanisms on tumor and metastasis inhibition are needed. While FuNPs with biodegradable ability can also act as drug carriers to achieve controlled drug release, exploring the mechanisms of FuNPs and discovering the ideal drugs to facilitate the synergistic combination for cancer therapy can open an avenue for a new class of therapeutic nanomedicines.

## Data Availability

The data presented in this study are available on request from the corresponding author.

## Supplementary Materials

The following are available online at https://www.mdpi.com/article/10.3390/pharmaceutics13121986/s1. Table S1a: Clinical chemistry values (mean ± SD) for male and female mice, Table S1b: Hematology values (mean ± SD) for male and female mice, Figure S1: High-dose fucoidan significantly impacts the liver and lymphatic organs. Graphs show that high-dose Fucoidan significantly increases the weight of the female spleen, the male liver and the thymus of both the male and female. All data are shown in means ± S.D. * *p* < 0.05; ** *p* <0.01 (two-tailed one-way ANOVA), Figure S2: The impact of a High-dose fucoidan to the thymus (**a**), GALT (**b**), mesenteric lymph node (**c**), and liver (**d**) is depicted in these representative microscopic images. (**a**) High-dose Fucoidan causes the corticomedullary ratio (black arrows) increase in the thymus. (**b**) High-dose Fucoidan also induces the follicular hyperplasia (grey circle) in the GALT. (**c**) Foamy microphages are shown (white arrows) in the mesenteric lymph nodes of the High-dose group. (**d**) The aggregation of foamy microphages (white arrow) and the apoptosis of the liver cells (black arrows) are presented 14 days after the high-dose Fucoidan injection. Scale bar = 100 μm for (**b**,**d**); 200 μm for (**a**) and 20 μm for (**c**) and Figure S3: Monitoring the internalization of L+S, Large, and Small FuNPs groups into 4T1 cells at 48 h incubation using fluorescent microscope. Scale bar = 50 μm.
