# Supplementary material for: Fucoidan-Based Nanoparticles with Inherently Therapeutic Efficacy for Cancer Treatment
Source: Pharmaceutics. 2021 Nov 23;13(12):1986. doi: 10.3390/pharmaceutics13121986 (PMC8707834; doi:10.3390/pharmaceutics13121986)
Supplement: Supplementary file 1 [file pharmaceutics-13-01986-s001.zip › pharmaceutics-1448862-supplementary.pdf]

Supplementary Materials: Fucoidan-Based Nanoparticles with Inherently Therapeutic Efficacy for Cancer Treatment

Chih-Sheng Chiang, Bo-Jie Huang, Jui-Yu Chen, Wee Wei Chieng, Seh Hong Lim, Wei Lee, Weoi-Cherng Shyu and Long-Bin Jeng

Table S1a. Clinical chemistry values (mean ± SD) for male and female mice.

Treatment	Control		Low-dose		Mid-dose		High-dose		Reference value	
Gender	♂	♀	♂	♀	♂	♀	♂	♀	♂	♀
AST (U/L)	82 ± 33	74 ± 16	69 ± 5	88 ± 48	85 ± 12	97 ± 36	62 ± 23	82 ± 15	42 - 106	52 - 112
ALT (U/L)	50 ± 30	30 ± 16	36 ± 8	38 ± 22	30 ± 7	42 ± 14	39 ± 5	40 ± 12	28 - 59	23 - 57
BUN (mg/dL)	21 ± 3	19 ± 3	20 ± 5	16 ± 6	23 ± 1	21 ± 3	22 ± 4	25 ± 6	16 -25	12 - 19
CREA (mg/dL)	0.1 ± -	<0.1 ± -	<0.1 ± -	<0.1 ± -	<0.1 ± -	<0.1 ± -	<0.1 ± -	<0.1 ± -	0.1 - 0.4	0.2 - 0.4
TBIL (mg/dL)	0.2 ± 0.1	0.2 ± 0.0	0.5 ± 0.2	0.2 ± 0.1	0.4 ± 0.2	0.4 ± 0.1	0.2 ± 0.1	0.2 ± 0.2	0.19 - 0.33	0.18 - 0.3
CK (U/L)	166 ± 85	146 ± 23	121 ± 36	166 ± 51	193 ± 38	222 ± 127	113 ± 40	233 ± 79	67 - 383	53 - 233
Albumin (g/dL)	2.2 ± 0.3	2.2 ± 0.0	2.2 ± 0.1	2.0 ± 0.1	2.1 ± 0.1	2.4 ± 0.1	2.0 ± 0.1	2.2 ± 0.2	3.2 - 3.9	3.6 - 4.3
Total protein (g/dL)	5.4 ± 1.3	5.0 ± 0.1	5.2 ± 0.1	4.7 ± 0.1	5.2 ± 0.1	5.2 ± 0.2	4.7 ± 0.3	4.8 ± 0.4	5.1 - 6.3	5.0 - 6.2
A/G ratio	0.7 ± 0.1	0.8 ± 0.1	0.7 ± 0.0	0.8 ± 0.1	0.7 ± 0.0	0.9 ± 0.1	0.7 ± 0.0	0.8 ± 0.0	0.8 - 1.9	1.0 - 2.7

Table S1b. Hematology values (mean ± SD) for male and female mice.

Treatment	Control		Low-dose		Mid-dose		High-dose		Reference value	
Gender	♂	♀	♂	♀	♂	♀	♂	♀	♂	♀
RBC (M/uL)	7.56 ± 0.61	8.09 ± 0.32	7.96 ± 0.74	8.43 ± 0.23	8.77 ± 0.53	8.47 ± 0.34	7.83 ± 0.27	8.18 ± 0.31	8.7 - 10.26	8.84 - 10.14
HCT (%)	36.2 ± 3.3	39.9 ± 2.3	37.9 ± 2.9	40. ± 0.8	42.9 ± 2.3	41.9 ± 1.4	39.6 ± 1.1	41.1 ± 1.0	41.4 - 47.6	42.5 - 48.9
HGB (g/dL)	12.4 ± 0.7	13.3 ± 0.8	12.9 ± 0.9	13.7± 0.4	14.2 ± 0.4	14.2 ± 0.3	13 ± 0.4	13.5 ± 0.7	14.4 - 16.4	14.9 - 17
PLT (K/uL)	1138 ± 80	976 ± 138	1026 ± 135	844 ± 303	1003 ± 109	854 ± 261	1035 ± 91	865 ± 58	920 - 1578	484 - 1488
WBC (K/uL)	5.00 ± 2.56	3.47 ± 0.58	2.42 ± 1.2	5.45 ± 0.99	2.63 ± 0.9	3.45 ± 1.74	2.53 ± 0.85	3.17 ± 1.16	6.79 - 17.4	7.16 - 20.23
Neu (%)	14.7 ± 3.0	16.4 ± 3.5	17.7 ± 3.1	12.8 ± 1.3	19.4 ± 6.8	13.1 ± 2.5	17.3 ± 3.4	17.1 ± 1.9	11.19 - 14.4	11.59 - 17.8
Lym (%)	82.9 ± 2.8	78.6 ± 4.4	77.3 ± 2.9	83.0 ± 3.8	75.5 ± 6.6	82.7 ± 3.8	79.0 ± 3.3	79.4 ± 2.6	70.1 - 84.5	76.4 - 80.8
Mono (%)	0.8 ± 0.5	2.9 ± 0.7	2.4 ± 1.1	2.6 ± 2.3	1.4 ± 0.3	2.4 ± 1.3	1.4 ± 1.0	1.8 ± 0.7	0 - 2.24	0 - 1.88
Eos (%)	1.7 ± 0.6	1.9 ± 0.9	2.5 ± 0.6	1.4 ± 0.3	3.1 ± 1.3	1.6 ± 1.0	2.1 ± 0.3	1.7 ± 1.1	0 - 3.05	0 - 5.34
Baso (%)	0.0 ± 0.0	0.2 ± 0.2	0.1 ± 0.2	0.2 ± 0.3	0.6 ± 0.1	0.3 ± 0.5	0.4 ± 0.8	0.0 ± 0.0	0 - 0.57	0 - 0.64

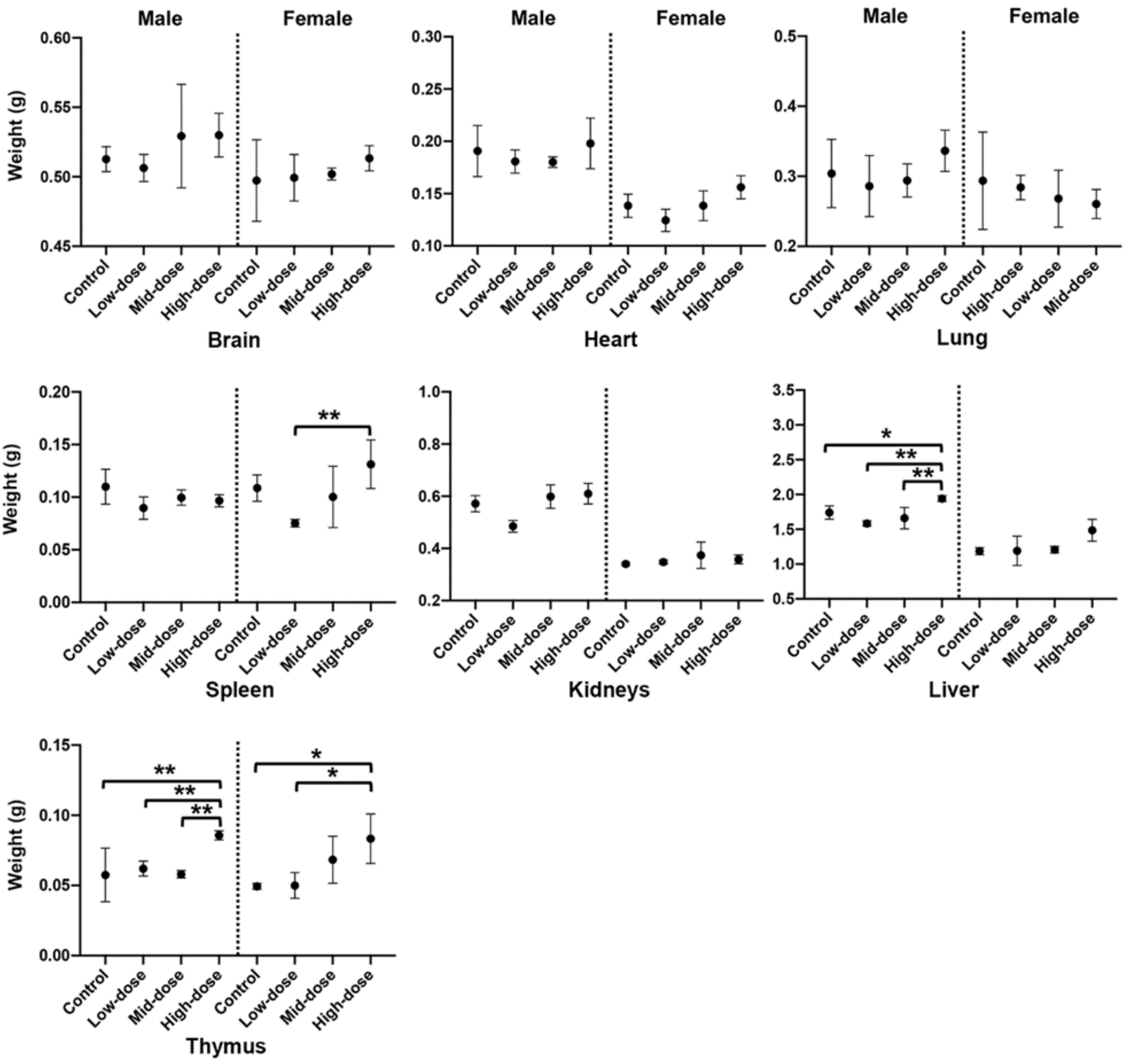

Figure S1. High-dose fucoidan significantly impacts the liver and lymphatic organs. Graphs show that high-dose Fucoidan significantly increases the weight of the female spleen, the male liver and the thymus of both the male and female. All data are shown in means ± S.D. * $p < 0.05$; ** $p < 0.01$ (two-tailed one-way ANOVA).

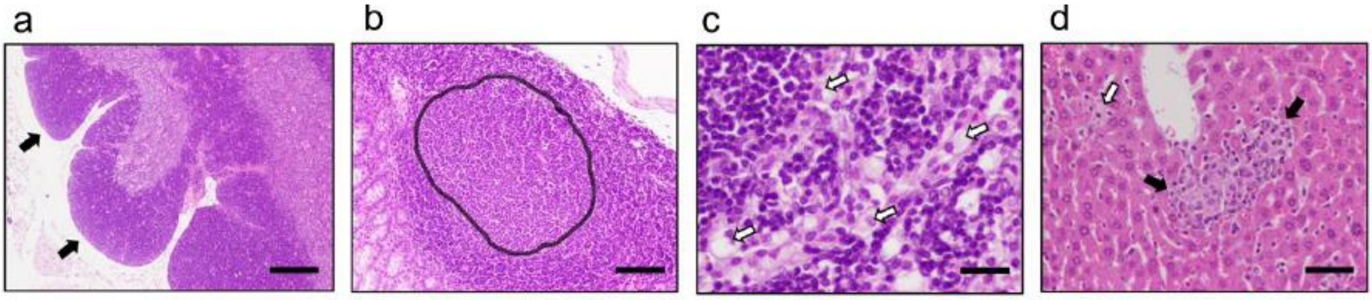

Figure S2. The impact of a High-dose fucoidan to the thymus (**a**), GALT (**b**), mesenteric lymph node (c), and liver (d) is depicted in these representative microscopic images. (a) High-dose Fucoidan causes the corticomedullary ratio (black arrows) increase in the thymus. (**b**) High-dose Fucoidan also induces the follicular hyperplasia (grey circle) in the GALT. (**c**) Foamy microphages are shown (white arrows) in the mesenteric lymph nodes of the High-dose group. (**d**) The aggregation of foamy microphages (white arrow) and the apoptosis of the liver cells (black arrows) are presented 14 days after the high-dose Fucoidan injection. Scale bar = 100 μm for (b and d); 200 μm for (**a**) and 20 μm for (**c**).

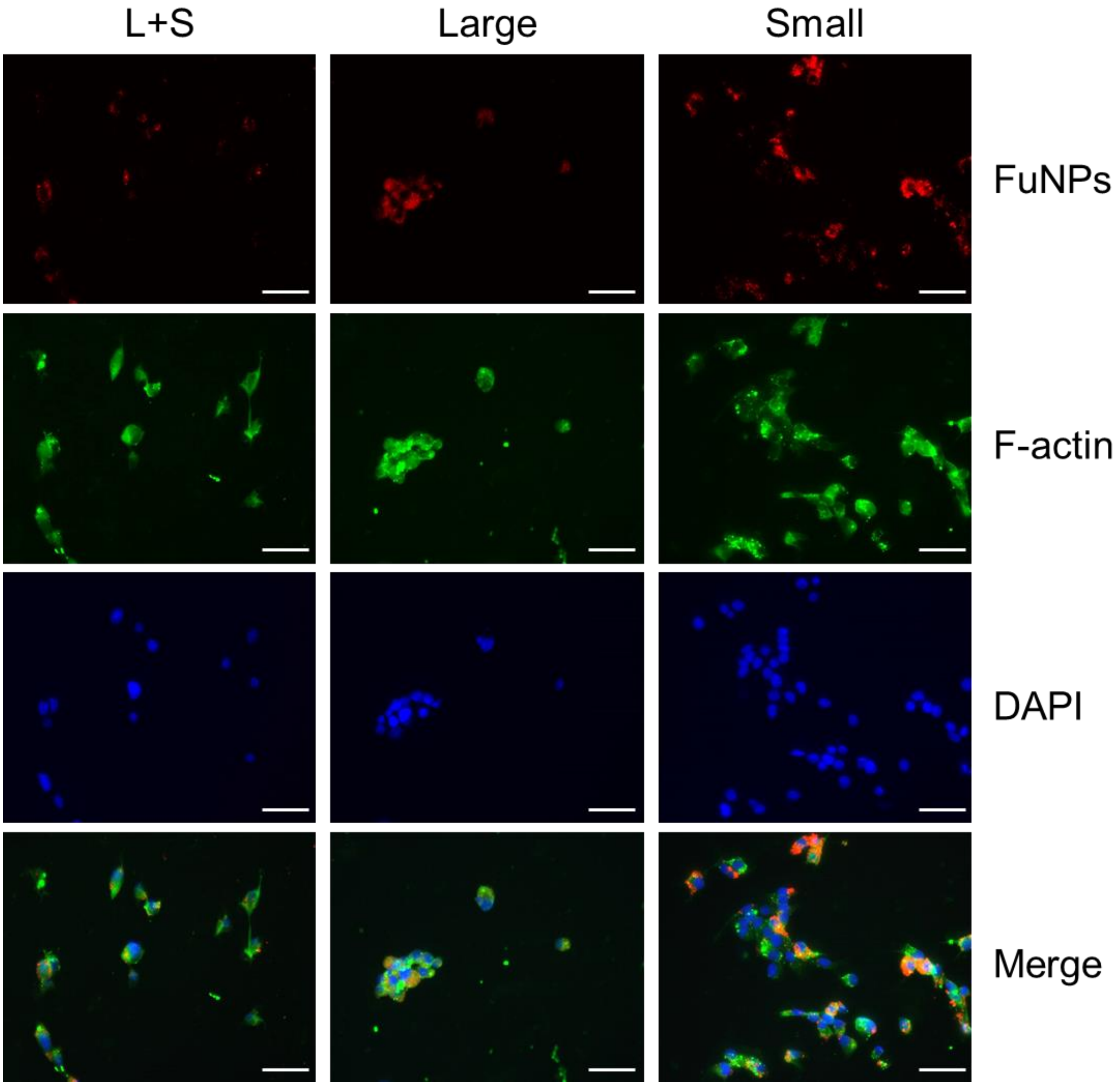

Figure S3. Monitoring the internalization of L+S, Large, and Small FuNPs groups into 4T1 cells at 48 h incubation using fluorescent microscope. Scale bar= 50 µm.
